# Solar Panel Corrosion: A Review

**DOI:** 10.3390/ijms26135960

**Published:** 2025-06-21

**Authors:** Zuraiz Rana, Pedro P. Zamora, Alvaro Soliz, Denet Soler, Víctor E. Reyes Cruz, José A. Cobos-Murcia, Felipe M. Galleguillos Madrid

**Affiliations:** 1 Centro de Desarrollo Energético de Antofagasta, Universidad de Antofagasta, Av. Universidad de Antofagasta 02800, Antofagasta 1271155, Chile; zuraiz.rana@ua.cl (Z.R.); denet.soler@uantof.cl (D.S.); 2 Departamento de Química y Biología, Facultad de Ciencias Naturales, Universidad de Atacama, Av. Copayapu 485, Copiapó 1530000, Chile; pedro.zamora@uda.cl; 3 Departamento de Ingeniería en Metalurgia, Facultad de Ingeniería, Universidad de Atacama, Av. Copayapu 485, Copiapó 1530000, Chile; alvaro.soliz@uda.cl; 4 Instituto de Ciencias Básicas e Ingeniería, Universidad Autónoma del Estado de Hidalgo, Carr. Pachuca—Tulancingo km. 4.5, Mineral de la Reforma 42184, Mexico; reyescruz16@yahoo.com (V.E.R.C.); jose_cobos@uaeh.edu.mx (J.A.C.-M.)

**Keywords:** corrosion, photovoltaic, material degradation, density functional theory, surface

## Abstract

The corrosion within photovoltaic (PV) systems has become a critical challenge to address, significantly affecting the efficiency of solar-to-electric energy conversion, longevity, and economic viability. This review provides a comprehensive analysis of electrochemical corrosion mechanisms affecting solar panels and environmental factors that accelerate material degradation, including (i) humidity, (ii) temperature fluctuations, (iii) ultraviolet radiation, and (iv) exposure to saline environments, leading to reduced performance and premature failures. The role of encapsulation materials, solder interconnections, and conductive coatings in the corrosion formation process is examined. Various electrochemical and surface characterization techniques provide insights into material degradation and corrosion mechanisms within panels. Essential parameters are presented and discussed, including materials used, geographical location of analysis, environmental considerations, and corrosion characterization techniques, to enhance the assessment of solar panels. This review emphasizes the importance of corrosion management for sustainable PV systems and proposes future research directions for developing more durable materials and advanced coatings.

## 1. Introduction

Solar energy has emerged as a promising renewable energy source, offering a sustainable alternative to traditional fossil fuels. Solar panels, also known as photovoltaic (PV) modules, play a central role in harnessing sunlight and converting it into electricity. As solar energy installations proliferate worldwide, ensuring solar panels’ long-term efficiency and performance becomes critical. One of the key challenges in this detection is solar panel corrosion, a complex process driven by various degradation mechanisms. Investigating solar panel corrosion mechanisms is extremely important to ensure solar panels’ longevity and sustained performance for several key reasons. (i) Preservation of energy output: solar panels generate electricity by converting sunlight into usable energy [1,2,3,4]. Corrosion can compromise the efficiency of solar cells by obstructing light absorption and reducing energy conversion efficiency [5,6,7]. By understanding the corrosion mechanisms, measures can be taken to prevent or mitigate its effects, ensuring optimal energy output over the panel’s operational life. (ii) Prolonging panel lifespan: solar panels are a significant investment, and their operational lifespan directly impacts the return on investment for solar energy projects. Corrosion-induced degradation can lead to premature failure of components and reduce the overall life expectancy of the panels. By investigating corrosion mechanisms, manufacturers and operators can design and implement measures to extend the panel’s service life, maximizing the economic benefits of solar energy installations. (iii) Maintaining system reliability: solar panels are often installed in remote or hard-to-reach locations, making regular maintenance challenging [8,9,10,11,12]. Corrosion can compromise the structural integrity of panels, leading to mechanical failures or electrical malfunctions. Investigating corrosion mechanisms helps identify vulnerable areas, enabling proactive maintenance and reducing the risk of unexpected system failures. (iv) Ensuring safety: corrosion-induced electrical or mechanical failures can pose safety hazards for personnel and the surrounding environment. For example, electrical contact corrosion might result in electrical arcing or fires. By understanding the corrosion mechanisms, appropriate safety measures can be implemented to minimize risks and ensure safe operation throughout the panel’s lifespan. (v) Improving design and materials: engineers and manufacturers can develop corrosion-resistant materials and optimize panel designs by studying specific corrosion mechanisms affecting solar panels. This results in solar panels better suited to withstand the effects of environmental exposure and harsh conditions, ultimately leading to more reliable and durable systems. (vi) Cost savings: corrosion-related failures can lead to unexpected repair or replacement costs. Investigating corrosion mechanisms enables the implementation of preventive measures, reducing maintenance expenses and avoiding costly downtime. Additionally, improved panel efficiency due to corrosion mitigation can result in higher energy yields and greater financial returns on solar investments [13,14,15,16]. Before delving into corrosion mechanisms, understanding the composition of solar panels is essential. PV modules typically consist of silicon-based solar cells, encapsulant materials (EVA or PVB), a back sheet, and a frame. The solar cells are protected by encapsulant and sandwiched between a back sheet and a front glass cover [17,18]. The frame provides structural support and protection for the entire assembly. Each component’s material properties and interactions become crucial factors in determining the vulnerability of solar panels to corrosion.

## 2. Corrosion Mechanisms and Degradation Models

**Figure 1:**
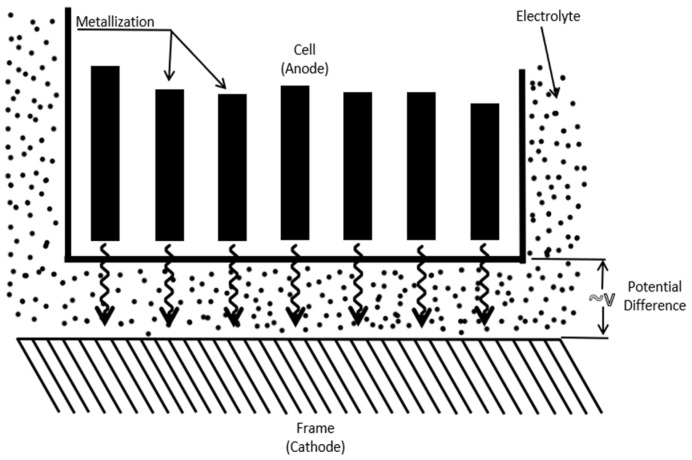
The mechanism of electrochemical corrosion.

Electrochemical corrosion is the most common and insidious degradation process affecting solar panels. It involves redox reactions between solar cell’s metal contacts and the surrounding environment. Moisture, humidity, and temperature fluctuations contribute to the formation of localized electrochemical cells on solar cell surfaces [19]. Over time, these cells lead to corrosion, causing pitting, etching, or general material deterioration. Electrochemical corrosion can significantly reduce solar cell’s light absorption and energy conversion efficiency, impacting the overall performance of PV modules. Figure 1, modified from previous work [20], shows the electrochemical corrosion mechanism.

### 2.1. Ohm’s Law Model

The charge transfer was calculated using a model based on Ohm’s law, as indicated in the equation that follows [20,21]:

(1) $$Q=\tau \cdot V\cdot k\cdot \int_{0}^{\theta_{0}}\frac{t}{l_{y}}\mathrm{dy}=\tau \cdot V\cdot k\cdot t\cdot \frac{2}{M+2}\cdot \left[\frac{M}{L}\cdot \tan^{-1}\left(\frac{1}{L}\tan\left(\frac{\theta_{0}}{4}\right)\right)-\left(M+1\right)\cdot \frac{\theta_{0}}{4}\right]$$

**Figure 2:**
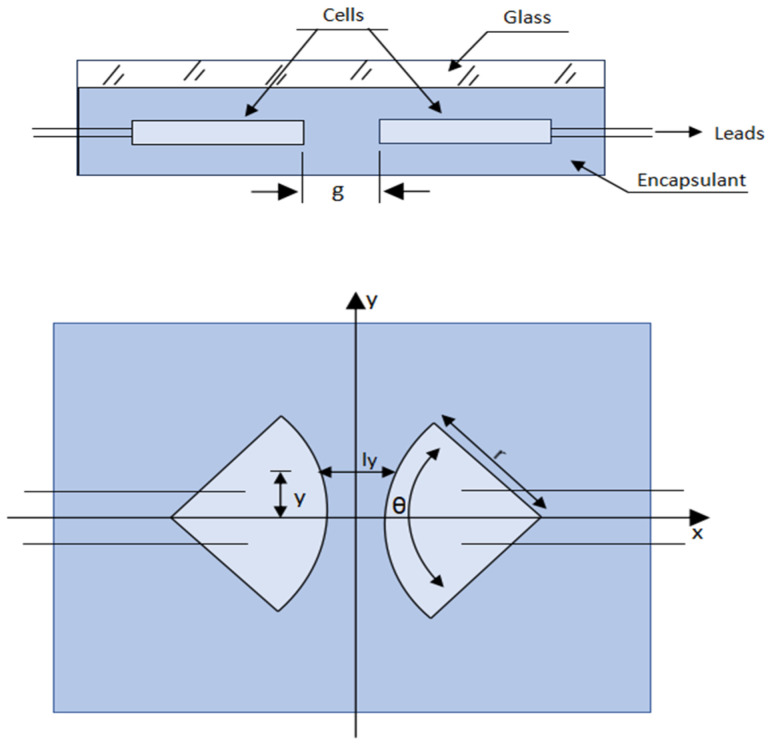
Geometry of electrochemical corrosion.

Consider L and M, such as:

(2) $$L=\sqrt{\frac{M-1}{M+1}}$$

(3) $$M=1+\frac{g}{2r}$$

where Q is the charge transfer (C), τ  time of exposition (h), V is the potential applied (V), k is the insulation electrical conductivity (Ω^−1^ cm^−1^), t is the effective electromigration conductivity (cm), and ly is the cell-to-cell distance at point y (cm), and Figure 2 altered from prior study [20], which is used for definitions of variables g, r, and $\theta_{0}$, respectively.

### 2.2. Pan Model

The proposed degradation model of the PV module output power is given by [22,23].

(4) $$D\left(t\right)=1-e^{\left(-{\mathrm{bt}}^{a}\right)}$$

where a and b are the parameters of the degradation model, and the constants a and b depend on the degradation mode considered. Equation (4) for corrosion and discoloration is expressed as follows:

(5) $$D_{\mathrm{Corr}}\left(t\right)=1-e^{\left(-b_{\mathrm{Corr}}t^{a_{\mathrm{Corr}}}\right)}$$

(6) $$D_{\mathrm{Dis}}\left(t\right)=1-e^{\left(-b_{\mathrm{Dis}}t^{a_{\mathrm{Dis}}}\right)}$$

where a (a_corr_ and a_dis)_ and b (b_corr_ and b_dis_) are parameters of the degradation model (a = 3.0868 and b = 5762.1012, allowing to track the evolution of the degradation D(t)).

The total degradation of PV module is estimated as follows:

(7) $$D_{\mathrm{PVM}}\left(t\right)=1-\prod_{i=1}^{n}\left(1-D_{i}\left(t\right)\right)$$

where n is the number of degradation modes, degradation model variables a and b are obtained by accelerated testing, and corrosion test conditions include temperature and humidity, major corrosion contributors.

Corrosion can induce degradation, which is influenced by temperature and humidity [24], both of which are exposure criteria in these experiments. Pan’s model mainly depends on experiments to identify the degradation parameters “a” and “b”. As a result, the precision and duration of the testing are limited.

This approach has some problems since these never occur in real-life situations. In fact, higher humidity and lower temperatures of solar modules were not encountered in the field, and the failure modes in long-term 85/85 testing were not encountered in the field. Depending on acceleration variables, a stronger 85/85 performance may not always indicate superior long-term performance in the field.

### 2.3. The Model of Exponential Development

An analytical model was developed to estimate the power attenuation of photovoltaic modules [25,26].

They posed the following assumptions.

- The module power (P) is a guide for performance evaluation.
- The degradation of solar cell modules is evaluated based on the initial power (P_0_).

The power of a photovoltaic module measured at a specific moment follows a Gaussian distribution. According to the probability, density is given by:

(8) $$P\left(p\right)=\frac{1}{\sqrt{2\pi \sigma }}e^{\left(-0.5{\left(\frac{p-\mu }{\sigma }\right)}^{2}\right)}$$

where P is the power module, l is the mean value, and μ is the standard deviation. The average power of the PV module decreases linearly with time. Thus, it can be calculated as follows:

(9) $$\mu \left(t\right)=P_{0}-A\cdot t$$

P_0_ is the average power at t = 0 (nominal power of component), A is a parameter reflecting an annual decline in component power, and t is time (in years). Of course, the validity of Equation (9) is limited by time (t) being smaller than P_0_/A. Another limitation arises from the assumption that A is constant over time. From Equation (9), it is simple to demonstrate that the power module decays at a steady rate relative to initial power for two consecutive years:

(10) $$\frac{\mu \left(n\right)-\mu \left(n-1\right)}{P_{0}}=-\frac{A}{P_{0}}$$

A/P_0_ (year^−1^) is the annual degradation rate. This linear degradation hypothesis is constrained by a lack of experience verifying this assumption in the literature. Based on studies on electrical component deterioration, we may assume that the degradation rate is exponential.

(11) $$\mu \left(t\right)=P_{0}\cdot e^{-\alpha t}$$

**Figure 3:**
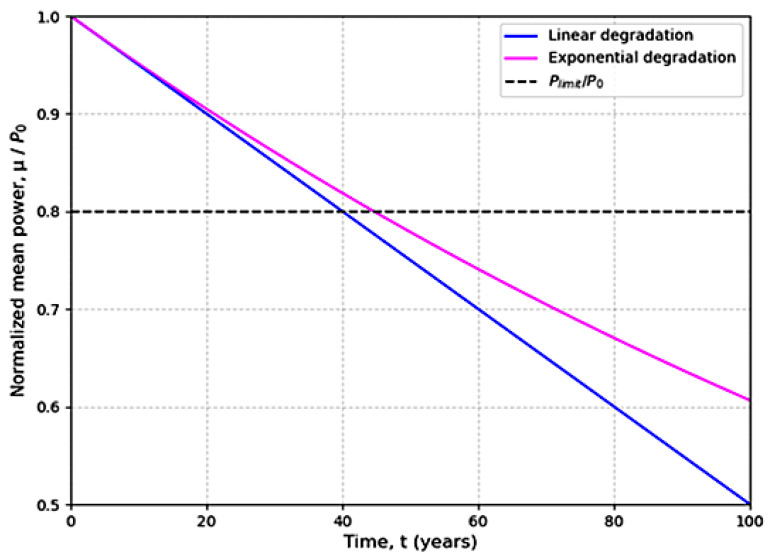
Linear and exponential power degradation rates for a 0–5% initial yearly degradation [25].

This model shows the degradation of photovoltaic modules during their lifetime (see Figure 3). However, many assumptions were made, and the results obtained do not necessarily reflect the reality of actual operating conditions.

### 2.4. Model Degradation by UV Stress

The leading cause of degradation of materials exposed to direct sunlight is photodegradation caused by ultraviolet rays. UVs are an essential factor in the degradation of photovoltaic modules, especially discoloration. The total dose of UV radiation can be considered as the number of photons absorbed by the degrading material, causing chemical changes. For PV modules, this degradation is reflected in changes in the transmittance of packaged components, resulting in a decrease in the current–voltage characteristics of PV modules. This reduction is quantified by the relative change in module short-circuit current. This represents the degradation factor given by:

(12) $$D\left(E\right)=\frac{I_{\mathrm{SC}}\left(E\right)}{I_{\mathrm{SC}}\left(E=0\right)}$$

where Isc(E) is the short-circuit current of the PV module, and E is the ultraviolet (UV) dose.

The degradation expression factor was reported by following equation [27].

(13) $$D\left(E\right)=\frac{\int_{\lambda_{\min}}^{\lambda_{\max}}\mathrm{SR}\left(\lambda \right)\cdot T_{\mathrm{cmx}}\left(\lambda \right)\cdot P\left(\lambda \right)\cdot T\left(\lambda ,E\right)d\lambda }{\int_{\lambda_{\min}}^{\lambda_{\max}}\mathrm{SR}\left(\lambda \right)\cdot T_{\mathrm{cmx}}\left(\lambda \right)\cdot P\left(\lambda \right)\cdot T_{0}\left(\lambda \right)d\lambda }$$

where SR(λ) is spectral response of PV cell, T(λ, E) and T_cmx_(λ) respectively represent the transmittances of the encapsulant and glass slide of PV module, T_0_(λ) is the transmittance of not irradiated PV cell. P(λ) is the spectral power density of the sun. [λ_min_, λ_max_] represents the integration interval for the wavelength range in which the spectral response of the PV cell is not zero.

For a photodegradation of encapsulant T(λ,E)/T_0_(λ) less than 70% in the range of [λ_min_, λ_max_], transmittance can be written as:

(14) $$T\left(\lambda ,E\right)=T_{0}\left(\lambda \right)\left[1-b_{\mathrm{cmx}}\left(\lambda \right)\cdot \ln\left(1+a_{\mathrm{cmx}}E\right)\right]$$

where a_cmx_ and b_cmx_ are parameters of material used for PV, and the relationship between UV dose E and exposure time (t) in solar spectrum P(λ) is:

(15) E=c·t

where

(16) $$c=\int_{0}^{400}T_{\mathrm{cmx}}\left(\lambda \right)\cdot P\left(\lambda \right)\cdot d\lambda$$

The integration extends to 400 nm, representing the practical limit of UV photodegradation. Combining Equations (10)–(13) and using the mean value theorem, Equation (14) is used to estimate the UV degradation of PV modules:

(17) $$D\left(t\right)=1-n\cdot \ln\left(1+a_{\mathrm{cmx}}\cdot c\cdot t\right)$$

where n = b_cmx_(λ), λ ϵ [λ_min_, λ_max_] and [λ_min_, λ_max_] represent integration interval in a wavelength range where solar cell spectral response is non-zero. This model presents a significant limitation. Its use requires understanding the specific properties of the materials used in PV cell production, such as a_cmx_, b_cmx_, SR(λ), T(λ, E), T_cmx_(λ), and T_0_(λ). The measurement of these properties requires rigorous equipment. Otherwise, the accuracy of the model is compromised.

### 2.5. Model Degradation by Temperature Stress

The Arrhenius equation is one of the most often used models for predicting temperature dependency in degradation processes [28,29]. For temperature-dependent processes, the Arrhenius method in Equation (15) can be used to forecast rate increase induced by a rise in temperature, as given by:

(18) $$K=A\cdot e^{-\frac{E_{a}}{\mathrm{RT}}}$$

(19) $$\frac{K_{1}}{K_{2}}=e^{\frac{E_{a}}{\mathrm{RT}}\left(\frac{1}{T_{2}}-\frac{1}{T_{1}}\right)}=A\cdot F_{T}$$

where K is the rate constant of the process, A refers to the former Arrhenius coefficient, E_a_ is apparent activation energy, R is gas constant, and T_1_ and T_2_ are sample temperatures. AF_T_ is a rate constant acceleration factor (ratio) of thermal degradation. To obtain these equations requires some assumptions and approximations:

- The rate constant K applies to a change in a property or performance. For example, if a material degrades due to color change or loss of mechanical properties, the rate constant of two degradation processes will likely differ.
- The entire temperature-dependent process leading to changes in performance (either in the presence or absence of light) follows Arrhenius dependence.
- The rate constant varies linearly with irradiance in the range of irradiances considered. This may not be the case for all polymers or at high irradiances, such as several times the maximum daytime irradiance near the equator. In such cases, a power law may apply to irradiance [30].
- The activation energy E_a_ of the entire temperature-dependent process (in the presence or absence of light) is constant in the considered temperature range.
- Weathering energy activation under accelerated conditions is only known for some materials. When a specific material’s activation energy is unavailable, it must be estimated from published activation energies for similar materials or determined from in-house experiments. For instance, the activation energy for the photochemical yellowing of EVA has not been published but can be estimated based on the E_a_ of photochemical yellowing established for various aromatic polymers [31].

The models based on Arrhenius have some limitations. Arrhenius equation can be used to quantify the effects of changes in temperature and irradiance on the rate of change of properties. However, it cannot provide a complete picture of the long-term degradation of PV modules because other stress factors or their combinations are also involved. These include humidity (causing physical and chemical processes and, in combination with temperature, producing mechanical stress), temperature cycling (causing thermomechanical stresses), and power generation (causing electrical and electrochemical stress-induced stresses), as well as other externally imposed stresses (wind, hail, pollutants, ocean air, sand, etc.).

### 2.6. Model Degradation Due to Temperature and Humidity Stress

The Peck model is an acceleration model that can consider temperature (T) and relative humidity (RH). The degradation rate prediction was performed using the Peck models, and their results were compared [32,33]. Escobar and Meeker [34] presented degradation models applicable to operating circumstances where temperature and humidity are accelerated stressors in a test, which is defined by the following relationship:

(20) $$\tau =A\cdot {\left(\mathrm{HR}\right)}^{n}e^{\left(-\frac{E_{a}}{k\cdot T}\right)}$$

where E_a_ is the effective activation energy of the degradation process, (k) is Boltzmann’s constant (8.617 × 10^−5^ eV/K), and A and n are two constants dependent on the failure mode. According to the Wiener process can be estimated as follows:

(21) $$\left(x_{i},Z_{\mathrm{ij}}\right)=1-e^{\left(-b^{0}\cdot \left(r\left(x_{i},\gamma \right)\cdot t_{\mathrm{ij}}\right)\right)}$$

In this case, x_1_ and x_2_ correspond to relative humidity (HR) and module temperature (T), refrence relative humidity (HR^0^) and temperature (T_0_) under reference conditions.

The acceleration factor is represented as:

(22) $$r\left(x_{1},x_{2},\gamma \right)=e^{\left(n\cdot \ln\left(\frac{\mathrm{HR}}{{\mathrm{HR}}^{0}}\right)-\frac{E_{a}}{k}\left(\frac{1}{T}-\frac{1}{T^{0}}\right)\right)}$$

where $\gamma =\left(n,E_{a}\right)$.

We consider that a^0^ = a for each stress level. The relation, according to Equation (21), becomes:

(23) $$\left(x_{i},Z_{\mathrm{ij}}\right)=1-e^{\left(-b\left(x_{i},\gamma \right)\cdot t_{\mathrm{ij}}^{a}\right)}$$

where

(24) $$b\left(x_{i},\gamma \right)=b^{0}\cdot e^{\left(a\cdot n\cdot \ln\left(\frac{\mathrm{HR}}{{\mathrm{HR}}^{0}}\right)-\frac{{\mathrm{aE}}_{a}}{k}\left(\frac{1}{T}-\frac{1}{T^{0}}\right)\right)}$$

The acceleration factor is defined by relation (22), and the equivalent temperature T_eq_ can be estimated, which represents degradation that would occur if modules were aged at a constant temperature over the same time period. T_eq_ can be calculated using the following relationship: [35]

(25) $$e^{\left(\frac{-E_{a}}{k\cdot T_{\mathrm{eq}}}\right)}=\frac{1}{t_{2}-t_{1}}\int_{t_{1}}^{t_{2}}e^{\left(\frac{-E_{a}}{k\cdot T_{\mathrm{module}}\left(t\right)}\right)}\mathrm{dt}$$

where t is time, T_module_(t) is time-dependent module temperature, and t_1_ and t_2_ are integration start and end times. Using the same method as shown below, the relative humidity equivalent H_eq_ can be estimated as:

(26) $${\left(H_{\mathrm{eq}}\right)}^{n}=\frac{1}{t_{2}-t_{1}}\int_{t_{1}}^{t_{2}}{\left(H\left(t\right)\right)}^{n}\mathrm{dt}$$

where t is time, H(t) is time-dependent ambient relative humidity, and t_1_ and t_2_ are integration start and end times.

Different types of corrosion mechanisms are visualized, but the most important is the galvanic corrosion [13,19,20]. This type of corrosion arises when two dissimilar materials come into electrical contact in the presence of an electrolyte. Solar panels are composed of various metals, and when in contact, they form galvanic couples. In the presence of moisture or humidity, these galvanic couples induce accelerated corrosion at the less noble metal’s expense. For example, when Al frames come into contact with copper or silver contacts, aluminum (Al) is more susceptible to corrosion. This form of corrosion poses a significant risk to the structural integrity and electrical performance of solar panels. Environmental factors can accelerate the corrosion processes in solar panels. Panels in coastal or industrial areas are more vulnerable due to exposure to saltwater, pollutants, and airborne debris. Saltwater corrosion can be particularly problematic, leading to the degradation of metal components and potential electrical failures. Moreover, sunlight’s ultraviolet (UV) radiation can initiate photochemical reactions that exacerbate corrosion. Crevice corrosion occurs in confined spaces or crevices between different components of the solar panel assembly. These crevices trap moisture and pollutants, creating localized environments conducive to corrosion. The interface between the solar cell and the encapsulant or the backsheet is a common location for crevice corrosion. Over time, corrosion spreads, compromising the panel’s integrity and, potentially, leading to catastrophic failure.

### 2.7. Degradation Mechanisms of Perovskite Solar Cells

**Figure 4:**
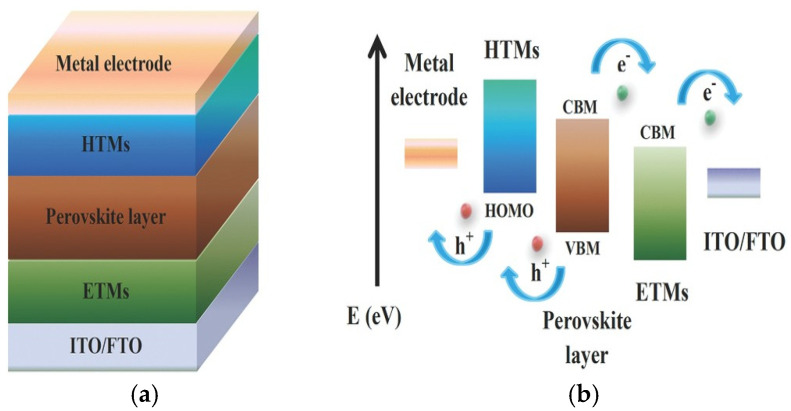
(**a**) A schematic diagram of general device configuration. (**b**) The work principle of normal perovskite solar cells [37].

Perovskite solar cells (PSCs) have emerged as a possible alternative to traditional silicon-based photovoltaics due to their high efficiency, ease of manufacturing [36], and low cost of manufacture. However, their stability and long-term performance remain significant issues [29]. Understanding the degradation mechanisms of PSCs is critical for increasing their economic value. Figure 4 shows the schematic diagram and working principles of perovskite solar cells.

#### 2.7.1. Moisture Sensitivity of PSCs

Moisture is a major contributor to PSC degradation. Moisture infiltration can lead to the degradation of the perovskite material, triggering phase transitions and the emergence of non-perovskite phases, ultimately causing a considerable decline in device efficiency. The presence of oxygen and light significantly speeds up this degradation process.

#### 2.7.2. Thermal Instability of PSCs

Perovskite materials exhibit considerable thermal instability, especially those derived from organic–inorganic lead halides. Extended exposure to elevated temperatures can lead to the breakdown of the perovskite structure, mainly affecting the lead and halide constituents. The thermal degradation results in diminished light absorption and charge transport properties, directly impacting the device’s efficiency.

#### 2.7.3. Light-Induced Degradation of PSCs

Photovoltaic cells can experience light-induced degradation, often termed “light soaking”, characterized by decreased efficiency when exposed to light. This phenomenon arises from the photochemical instability of the perovskite material, especially concerning the loss of halide ions or phase transitions when subjected to extended light exposure. The effects of light-induced degradation become significantly more evident when exposed to high-intensity illumination and elevated temperatures.

#### 2.7.4. Ion Migration of PSCs

Perovskite materials demonstrate notable ionic migration, which can potentially result in defect formation, loss of perovskite phases, and overall device degradation. The movement of halide and metal ions, including lead ions, can create non-active areas, which may considerably diminish the device’s efficiency and stability.

## 3. Effects of Corrosion on Solar Panel Performance

The consequences of solar panel corrosion are multifaceted and directly impact their performance and lifespan. The reduction of short-circuit current was attributed to optical transmission losses in discolored encapsulants above solar cells. An increase in series resistance was generally associated with electrochemical corrosion and/or debonding of cell metallization. Decreased shunt resistance from external current paths reduces power in silver-print and tri-metal cells, with short-circuit current losses also significantly impacting power in PVB-encapsulated samples [20].

The principal effect of corrosion evidence is as follows: (i) Loss of efficiency: corrosion-induced degradation on the surface of solar cells reduces their ability to absorb and convert sunlight into electricity. The formation of corrosion products and roughened surfaces obstructs light transmission and diminishes energy conversion efficiency. Gradual efficiency losses result in decreased power output, potentially reducing the overall energy yield of solar installations. (ii) Mechanical degradation: corrosion attacks structural components such as frames and backsheets, weakening their mechanical properties. Weakened frames may lead to mechanical failures due to wind or snow, such as bending or cracking. Moreover, backsheet degradation can cause delamination, exposing solar cells to environmental elements and accelerating their deterioration. (iii) Electrical performance: corrosion of electrical contacts can increase electrical resistance, leading to power losses and hotspots within the panel. Hotspots can further exacerbate the corrosion process, compromising the entire solar panel’s electrical performance and, in extreme cases, leading to module failure. The energy efficiency and longevity of photovoltaic (PV) modules are significantly affected by the environmental conditions of their operational sector, including humidity, ultraviolet (UV) radiation, and thermal stress (See Table 1). 

## 4. Corrosion Mechanism of Solder Interconnection

The oxidation potentials of Ag/Ag^+^, Cu/Cu^2+^, Pb/Pb^2+^, and Sn/Sn^2+^ are +0.799, +0.337, −0.126, and −0.136 V, respectively. Metals with lower corrosion potential corrode faster than metals with higher corrosion potentials [55]. As shown below, Sn and Pb materials act as anodes and tend to accelerate corrosion, while Ag and Cu materials act as cathodes within a corrosion cell.

### 4.1. The Cathodic Sub-Processes

Typically, cathodic reactions involved during the atmospheric corrosion process, depending on the environment, are:

#### 4.1.1. Hydrogen Evolution Reaction (HER)


(27) $$2H^{+}+2e^{-}\to H_{2}\;$$

(28) $$2H_{2}O+2e^{-}\to H_{2}+2{\mathrm{OH}}^{-}\;$$

#### 4.1.2. Oxygen Reduction Reaction (ORR)


(29) $$2H^{+}+\frac{1}{2}O_{2}+2e^{-}\to H_{2}O\;$$

(30) $$O_{2}+H_{2}O+4e^{-}\to 4{\left(\mathrm{OH}\right)}^{-}$$

### 4.2. The Anodic Sub-Processes

Metals with more negative potential values corrode faster than metals with more positive ones in a galvanic cell. Thus, in a metallic system composed of Ag, Cu, Pb, and Sn metals, the Sn and Pb materials become anodes and tend to corrode quickly according to:

(31) $$\mathrm{Sn}\to {\mathrm{Sn}}^{2+}+2e^{-}\;$$

(32) $$\mathrm{Pb}\to {\mathrm{Pb}}^{2+}+2e^{-}\;$$

**Figure 5:**
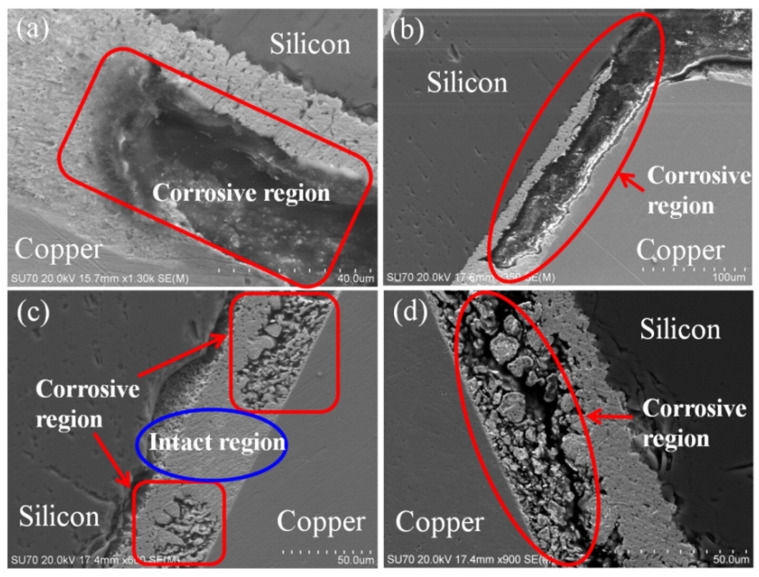
(**a**)The edge of the soldered connection corroded first. (**b**) Corrosion extended from the edge to the center at the contact with the Ag electrode. Water may easily cover the edge of a soldered connection, leading to galvanic corrosion. (**c**) Corrosion reached the center of the soldered connection. (**d**) The soldered connection is divided into two pieces along the interface [71].

Considering that the solar panel is exposed to a near-neutral pH media, the overall reactions for Pb and Sn are according to the following expressions. Furthermore, the microstructure of the soldered junction of cells was examined during the aging test (See Figure 5).

(33) $$\mathrm{Pb}+2H_{2}O\to {\mathrm{Pb}}^{2+}+H_{2}+2{\mathrm{OH}}^{-}\;$$

(34) $$2\mathrm{Pb}+O_{2}+2H_{2}O\to 2{\mathrm{Pb}}^{2+}+4\mathrm{OH}\;$$

(35) $${\mathrm{Pb}}^{2+}+2{\mathrm{OH}}^{-}\to \mathrm{Pb}{\left(\mathrm{OH}\right)}_{2}$$

(36) $$\mathrm{Sn}+2H_{2}O\to {\mathrm{Sn}}^{2+}+H_{2}+2OH^{-}\;$$

(37) $$2\mathrm{Sn}+O_{2}+2H_{2}O\to 2Sn^{2+}4{\mathrm{OH}}^{-}\;$$

(38) $${\mathrm{Sn}}^{2+}+2{\mathrm{OH}}^{-}\to \mathrm{Sn}{\left(\mathrm{OH}\right)}_{2}$$

On the other hand, cell degradation caused by moisture and heat during aging can lead to the formation of Ag_2_O. Oxidation of Ag leads to the formation of two-phase oxides. The reactions that form these oxides are shown in the diagram below.

(39) $$4\mathrm{Ag}+O_{2}\to 2{\mathrm{Ag}}_{2}O\;$$

(40) $$2\mathrm{Ag}+O_{2}\to 2\mathrm{AgO}\;$$

Ag can be thermodynamically oxidized by naturally occurring atmospheric oxygen to form Ag_2_O. The thickness of the formed Ag_2_O layer has been reported to be in the range of 10–20 Å. Therefore, it is considered that after the damp-heat test, an oxide having an Ag_2_O phase formed on the Ag surface, and its concentration increased.

**Figure 6:**
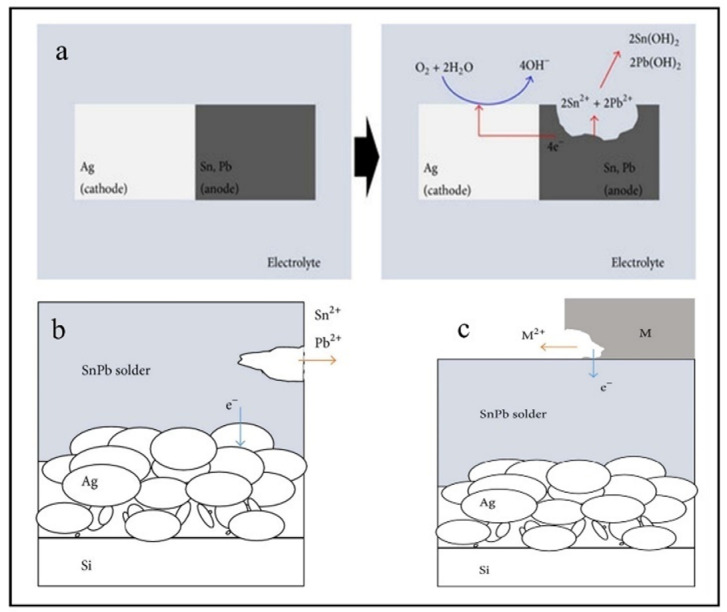
(**a**) The corrosion mechanism at the interface between the SnPb solder and Ag paste. A mitigation method for corrosion of SnPb solder: (**b**) corrosion of a sample without a sacrifice metal and (**c**) corrosion of a sample with a sacrificial metal anode (Zn or Al) [58].

Therefore, if a metal with a lower potential value than Sn or Pb is added to a corrosion battery system, it will act as an anode and be susceptible to corrosion. Zn/Zn^2+^ and Al/Al^3+^ oxidation potential values are −0.763 V and −1.662 V, respectively. Zn and Al, when added to photovoltaic soldering systems, have higher corrosion rates than Sn and Pb shown in Figure 6.

**Figure 7:**
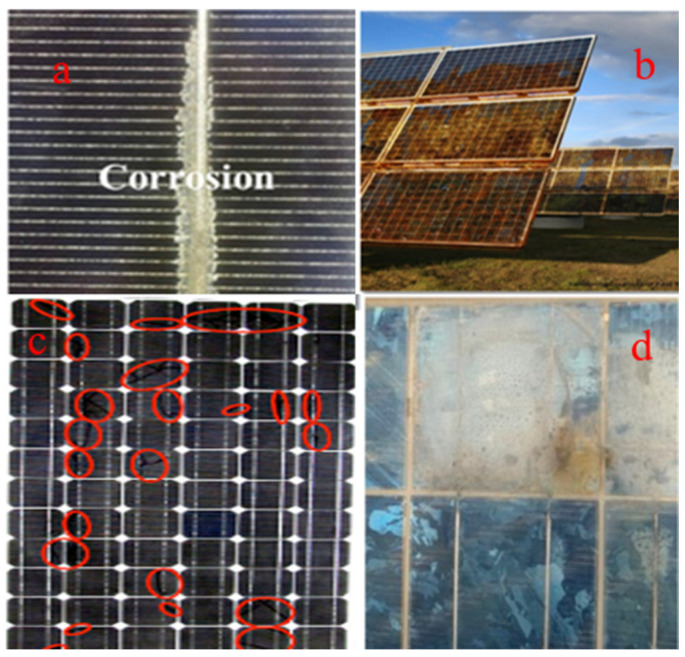
(**a**) Corrosion in the silicon solar cell. The gas bubbles can grow and merge, increasing delamination. (**b**) Corrosion in solar panels due to electrochemical processes. (**c**) The red circles show corrosion within solar panels due to cracks [128]. (**d**) Moisture and corrosion in modules [116].

The photovoltaic effect is the generation of voltage or electric current in a substance when exposed to light. Electrons are considered charge carriers for electric current because light stimulates their transfer into a higher energy band [20]. As seen in Figure 7, corrosion initially develops on the solar module’s edge due to moisture and its interaction with sodium in the cover glass. Transparent conductive oxide (TCO) or glass cover corrosion is irreversible. It causes ongoing power outages [118]. This operation will cause TCO to become milky and lose its conducting qualities, lowering the solar module’s efficiency.

## 5. Perspectives in Mitigation Related to Corrosion Management

### 5.1. Material Selection and Coatings

Using corrosion-resistant materials for solar panel construction is crucial for reducing vulnerability to corrosion [58]. Stainless steel or corrosion-resistant aluminum alloys for frames and conductive materials with protective coatings for electrical contacts can significantly prolong the panel’s lifespan.

### 5.2. Design Improvements

Design features that minimize moisture ingress, promote proper drainage, and reduce crevice formation can mitigate the potential for corrosion. Encapsulation techniques with improved water barrier properties can also enhance the long-term performance of solar panels.

### 5.3. Environmental Considerations

Locating solar installations away from highly corrosive environments, such as coastal regions, can reduce exposure to saltwater and other corrosive substances. Implementing protective measures, such as UV-resistant coatings, can combat the effects of UV radiation on the panel’s surfaces.

### 5.4. Monitoring and Maintenance

Regular monitoring and maintenance are vital to identifying early signs of corrosion and addressing them promptly. Periodic inspections and cleaning to remove accumulated debris and pollutants can help prevent the onset and progression of corrosion.

## 6. Critical Analysis

Understanding and reducing photovoltaic (PV) system degradation mechanisms is essential to optimizing energy generation and reducing maintenance costs as demand for solar energy rises globally. Materials such as Sn, Pb, Cu, and Ag are commonly used to make solder interconnections in solar panels since they have significance for maintaining chemical stability and electrical conductivity [55]. However, these materials are subject to corrosion, particularly when exposed to environmental stresses like humidity, temperature changes, and air pollution [52]. Material degradation caused by corrosion gradually compromises the electrical functionality and lifespan of solar panels.

Beyond generalization, model extrapolation capacity is critical for increasing prediction accuracy. More relevant data from a broader variety of environmental variables must be collected to improve the accuracy of solar panel corrosion forecasts. For example, databases should contain samples from diverse geographical regions and weather conditions, particularly those with high humidity or salt exposure, as these conditions substantially impact corrosion rates. Furthermore, research conducted under controlled laboratory circumstances may not adequately represent real-world scenarios. Predictive models should be validated using field samples to determine their applicability in various contexts. Furthermore, data-collecting procedures, such as electrochemical impedance spectroscopy, should be carefully described to guarantee that results are reproducible and reliable.

In addition to the natural corrosion process [7], external factors like temperature and humidity enhance the breakdown of solar cells. For example, in hot and humid conditions, Ag oxidizes and forms a thin coating of Ag_2_O. Although this oxide layer is rather thin, it affects panel conductivity and overall performance. Furthermore, in very corrosive conditions (coastal locations exposed to salt or industrial districts with high levels of air pollution), Sn, Pb, Cu, and Ag corrosion rates are much greater. Understanding these degradation pathways is crucial for creating more lasting solar systems that tolerate various environmental conditions.

## 7. Typical Electrochemical and Surface Characterization Techniques

Electrochemical and surface characterization techniques are essential for evaluating the performance and effectiveness of eco-friendly corrosion inhibitors. These approaches provide critical information on inhibitory characteristics of organic compounds derived from plant sources, with insights into their interactions with metal surfaces in corrosive conditions. The following is a list of common electrochemical and surface characterization techniques for assessing corrosion inhibitors.

### 7.1. Electroluminescence Imaging Analysis (EL)

**Figure 8:**
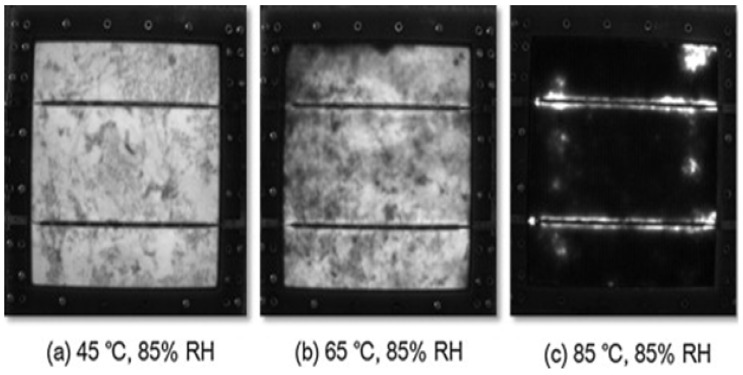
EL image of sample after DH tests [55].

Figure 8 depicts the degeneration of a PV module using an EL image after 3500 h of aging at 45/85, 65/85, and 85/85. EL pictures may identify flaws in cells or places where the electricity of excitation cannot reach. The black picture indicates a problem with the PV module generating current. The findings might be due to solar cell failure, electrode degradation, or solder connector corrosion. Figure 6 shows that the PV module under 85/85 was substantially deteriorated.

### 7.2. Scanning Electron Microscopy (SEM), Energy Dispersive Spectroscopy (EDS) Analysis

**Figure 9:**
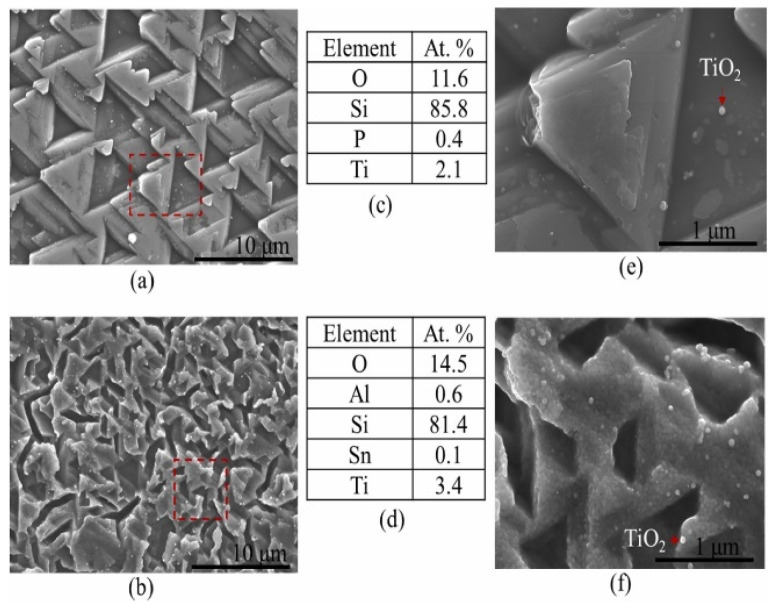
SEM and EDS analyses of a solar cell extracted from the edge of PV Module X. (**a**,**b**) SEM micrographs and (**c**,**d**) EDS analyses acquired ca. 10 mm from the edge and just at the edge of a solar cell. SEM micrographs of marked-out areas in (**a**,**b**) are shown in (**e**,**f**), respectively. The EDS analyses are represented in atomic % (at. %) [123].

SEM-EDS equipment is the pinnacle of technological skill, providing unprecedented specificity and power in material surface characterization. It is a fundamental technique for investigating materials, particularly metals that corrode. These photos, acquired at extremely high resolution, offer valuable windows into the complex world of corrosion processes. Beyond mere observation, SEM-EDS helps decipher the surface morphology and elemental distribution in the layer of the formed corrosion products (see Figure 9). This reveals the complex interaction of molecule adsorption, demonstrating that the development and adherence of thin organic coatings are critical for corrosion inhibition. Examining the depths of these images allows us to obtain insight into mechanisms that regulate the deterioration or preservation of metal surfaces. Consequently, SEM-EDS is more than just a tool; it is also a means of understanding and modifying the fate of materials in their struggle against corrosion. At the same time, the combined energy dispersive analysis technique allows us to extract a more comprehensive set of data, revealing insight into the precise chemical composition of these critical corrosion barriers [128].

The SEM micrographs and EDS analyses in Figure 9 reveal significant differences in microcrystalline structures and elemental compositions of a solar cell, particularly at the edges. The EDS data show higher oxygen levels at edges, indicating degradation due to moisture ingress. This degradation leads to poor charge carrier generation and high parasitic recombination, contributing to the observed decline in maximum power output (P_max_) of field-aged PV modules. The presence of phosphorus suggests it originates from the EVA encapsulation, while aluminum and tin detected at the edges indicate leaching and migration due to moisture. These findings highlight environmental factors’ impact on solar cells’ performance and longevity.

### 7.3. X-Ray Photoelectron Spectroscopy Analysis (XPS)

**Figure 10:**
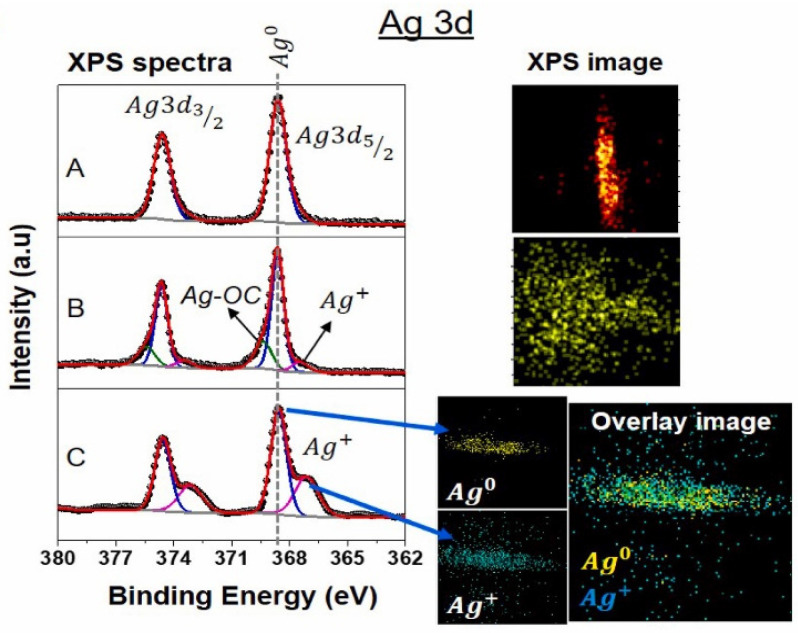
XPS spectral lines of Ag 3d and corresponding XPS image/mapping of control and degraded samples. The degraded multicrystalline Al-BSF sample indicates higher oxidation of Ag (Ag^+^) compared to the PERC sample. The atomic % profile of Ag and Ag^+^ was generated from XPS imaging, and the corresponding overlay image shows the locations of Ag and Ag^+^ [113].

Figure 10 shows an XPS analysis of the Ag metal finger. The image below exhibits high-resolution Ag 3d XPS spectra for each sample after the last stage of depth profiling. The deconvolution of peaks shows that Ag is oxidized in damaged samples [129,130]. It is also clear that multicrystalline materials have a faster Ag oxidation rate than monocrystalline materials. The control sample appears to have no oxidation, as expected. We also took XPS images of the whole spectrum for each sample. The control sample shows the presence of metallic Ag on its surface. The extensively oxidized multicrystalline sample shows two deconvoluted peaks: Ag (368.6 eV) and Ag^+^ (367.13 eV).

The degraded PERC sample displays a new peak (≈369 eV) for Ag-OC bonding, showing an oxidized Ag surface associated with C-O species. The atomic% profile of the Ag 3D images was recovered, and the positions of Ag and Ag^+^ were calculated in the damaged Al-BSF sample. Ag overlay mapping reveals the presence of metallic and oxidized Ag on the grid line. This shows that the gridline is corroding for both methods, as shown in prior studies [131]. In other words, the resistivity of the finger conductor rises as Ag oxidizes. However, this is not the main cause of the increase in resistance. If this were the case, the EL image structure would be darker along the finger’s path and brighter at busbars, reaching a minimum around midway between busbars. The impact of backsheet WVTR on corrosion rate has also been considered; however, there is little difference between the two WVTRs, and EL pictures taken at each stage demonstrate that, for monocrystalline and multicrystalline modules, respectively, corrosion starts at the busbar after 2000 h of exposure. The multicrystalline modules with high WVTR backsheets would have darkened earlier than the monocrystalline samples if the difference in WVTRs had a significant impact on the rate of DH degradation.

## 8. Organic Solar Cells (OSC)

**Figure 11:**
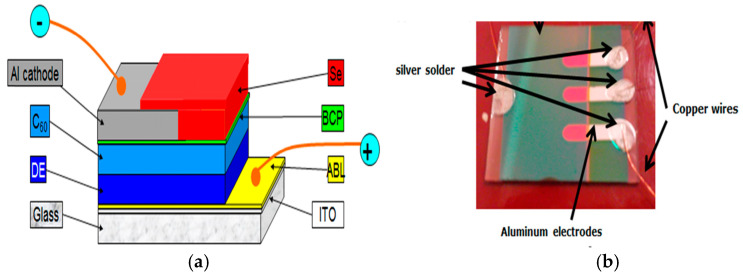
(**a**) A multilayer organic solar cell architecture ITO (indium tin oxide), ABL (anode buffer layer), DE (electron donor), C60 (electron acceptor), BCP (exciton blocking layer), Al cathode (aluminum electrode), Se (selenium). (**b**) A fabricated organic solar cell.

Organic solar cells (OSCs) offer a promising alternative to photovoltaic energy, providing a more flexible, lightweight, and potentially cost-effective option compared to traditional silicon-based solar cells [132]. Organic semiconductor cells, made from polymers and carbon-based molecules, generate electricity from sunlight through the photovoltaic effect and are sustainably manufactured on flexible substrates. Within their manufacturing process, there are two main types of OSC architecture. Multilayer cells feature donors and acceptor layers deposited on the other, making fabrication easier (Figure 11) and more reproducible. However, this design reduces contact area, which limits charge carrier mobility. In contrast, bulk heterojunction cells (interpenetrated structures) deposit donor and acceptor materials together as a blended mixture. This design improves charge carrier mobility from donor to acceptor, increasing efficiency. However, the fabrication technique is more complex, and reproducibility can sometimes be challenging [133].

The cell’s layers may be coated on flexible substrates. Organic solar cells (OSCs) were constructed using a typical device architecture of ITO/PEDOT: PSS. The former has lower light transmittance than ITO but is still commonly used in fabrication. Although this type of solar cell is significantly more affordable than conventional silicon-based cells, its efficiency has reached 19% in recent years under laboratory conditions [134].

The primary challenge for OSCs remains their durability as organic molecules tend to degrade due to various environmental factors.

As seen in Figure 11a,b, the cell includes a protective selenium layer (shown in red) that prevents and delays active layer oxidation. Without the selenium layer, the cell would decompose within minutes [132].

### 8.1. Exposure to Light and Photo-Oxidation

One of the main degradation mechanisms in OSCs is photo-oxidation, a process in which UV radiation breaks chemical bonds in the cell’s layers (including a semiconductor material, acceptor layer, donor layer, and exciton-blocking layer). This generates free radicals that degrade the cell’s energy conversion efficiency. Prolonged exposure to light can also induce structural changes in semiconductor materials, reducing charge carrier mobility and impacting the stability of the device [135].

### 8.2. Influence of Oxygen and Humidity

Oxygen and humidity are two of the main degrading agents in OSCs. These elements can penetrate through protective layers and react with active materials, leading to the oxidation of electron donors and acceptors. Humidity can cause defects in the active layer, affecting charge transfer and reducing the cell’s performance [132] (Figure 11b).

### 8.3. Thermal Degradation

OSCs are sensitive to temperature as organic materials can undergo structural changes and degradation at high temperatures. For this reason, these materials are analyzed using thermogravimetric studies to determine the temperature at which decomposition begins [136].

### 8.4. Mechanical Degradation

Due to their flexibility, OSCs are exposed to mechanical stresses that can lead to the formation of cracks and defects in active layers. These imperfections can increase oxygen and humidity permeability, accelerating degradation [132,137].

### 8.5. Corrosion in Organic Solar Cells

Corrosion is another critical issue in OSCs, primarily affecting metal contacts and conductive materials.

#### 8.5.1. Electrode Corrosion

The electrodes in OSCs, typically made of metals such as silver (Ag), aluminum (Al), or indium tin oxide (ITO), can undergo corrosion due to exposure to humidity and oxygen. Silver, for instance, can form silver sulfide in the presence of sulfur compounds, reducing electrode conductivity and negatively impacting device efficiency [138].

#### 8.5.2. Galvanic Corrosion

When different metals are used in cell structures, galvanic corrosion can occur due to potential differences between materials. This phenomenon accelerates electrode degradation and shortens the device’s lifespan [138].

#### 8.5.3. Light-Induced Chemical Reactions

Some materials used in OSCs can undergo light-induced chemical reactions, which trigger the degradation of their molecular structure. This degradation results in a lower generation of charge carriers and reduced mobility, directly impacting the device’s performance. Over time, these reactions can cause significant changes in the morphology of active layers, leading to the formation of defects, phase separation, or crystallization of certain materials. Such structural modifications accelerate the corrosion process and increase electrical resistance, making it more difficult for charge carriers to move efficiently through the device. As a result, the overall efficiency of solar cells declines sharply, limiting their long-term viability [139].

## 9. Strategies to Mitigate Degradation and Corrosion

Various strategies have been developed to enhance the stability and lifespan of OSCs.

### 9.1. Use of High-Barrier Encapsulants

The development of high-barrier encapsulating materials is essential for improving the stability and longevity of OSCs by preventing infiltration of oxygen and moisture, two of the main factors responsible for their degradation. These external elements can rapidly oxidize active materials without proper encapsulation, leading to performance loss and a shorter device lifespan.

Different encapsulation approaches have been explored to enhance protection. Sealed glass enclosures provide excellent impermeability but can limit flexibility, a key advantage of OSCs. Metal oxide layers, such as aluminum oxide (Al_2_O_3_) or silicon oxide (SiO_2_), offer high resistance to gas diffusion while maintaining transparency and compatibility with flexible substrates. High-barrier polymer coatings, such as multilayer organic–inorganic hybrid films, combine flexibility with superior moisture and oxygen resistance, making them an ideal solution for large-scale applications.

By integrating these advanced encapsulation materials, OSCs can significantly improve their durability, efficiency, and commercial viability, making them more competitive in the renewable energy market [140].

### 9.2. The Search for New Semiconductor Materials

Improved photochemical and thermal stability is fundamental to extending the lifespan and efficiency of OSCs. While highly versatile and cost-effective, traditional organic materials are prone to degradation under prolonged exposure to light, heat, and environmental factors. Researchers are developing advanced polymers and organic molecules with more robust chemical structures to overcome these limitations.

One promising approach is the design of highly conjugated polymer backbones, which enhance the delocalization of charge carriers, making materials more resistant to photochemical breakdown. Additionally, non-fullerene acceptors (NFAs) have demonstrated superior stability to traditional fullerene-based counterparts, offering higher efficiency and longer operational lifetimes.

Another key innovation involves incorporating crosslinked and dopant-free materials, preventing phase separation and morphological instability over time. These materials reduce the formation of defects that can trap charges and hinder performance. Scientists are also exploring hybrid organic–inorganic systems, which combine the flexibility and lightweight properties of organic materials with the durability of inorganic counterparts, resulting in OSCs that are more resilient to thermal and oxidative stress.

By continuously improving molecular design and synthesis techniques, the development of stable materials is paving the way for longer-lasting, high-performance OSCs, making them a more viable and competitive option in the renewable energy sector [140].

### 9.3. Electrode Modification

One of the key challenges in OSC technology is the degradation of electrodes, which directly affects device efficiency and lifespan. The corrosion of metal contacts and transparent conductive layers due to exposure to oxygen, moisture, and other environmental factors leads to increased electrical resistance, reduced charge transport, and performance loss. To address this, researchers focus on modifying electrodes with more corrosion-resistant materials to enhance their durability and conductivity.

A widely used approach is improving transparent conductive oxides (TCOs), such as indium tin oxide (ITO), which are commonly employed as front electrodes in OSCs. However, ITO is susceptible to degradation under bending stress (in flexible devices) and can corrode over time. To mitigate this, alternative TCOs like doped zinc oxide (ZnO: Al) or ultrathin metal grids coated with protective layers are being developed to maintain transparency while improving resistance to environmental degradation.

Additionally, metallic back electrodes, often composed of silver (Ag) or aluminum (Al), are prone to oxidation and sulfurization, which reduces their conductivity. To counter this, researchers are incorporating stable metal alloys and thin passivation layers, such as gold (Au) nano-coatings or graphene-based protective films, which prevent chemical reactions while maintaining excellent charge extraction properties.

Another promising strategy involves using hybrid electrode structures, where conductive polymers or carbon-based materials (such as carbon nanotubes or graphene) are integrated into the electrode design. These materials provide higher flexibility and mechanical stability and offer improved chemical resistance, making them ideal for next-generation OSCs.

By integrating these advanced corrosion-resistant coatings, alternative TCOs, and hybrid electrode materials, OSCs can achieve greater efficiency, longer operational stability, and enhanced commercial viability [140].

### 9.4. Device Engineering

Advancements in device engineering play a crucial role in minimizing degradation and enhancing the performance of OSCs. By refining structural design and layer arrangement, researchers aim to improve charge transport, stability, and resistance to environmental factors.

One key approach is the optimization of active layer morphology, ensuring a finely tuned donor–acceptor domain distribution. A well-structured morphology enhances charge separation and transport, reducing recombination losses and increasing efficiency. Researchers achieve this by controlling solvent evaporation rates, annealing conditions, and molecular packing, which leads to a more stable and uniform film.

Additionally, advanced charge transport layers (CTLs) are being developed to enhance the extraction and movement of charge carriers. Materials such as self-assembled monolayers (SAMs) and low-defect metal oxides improve energy level alignment and minimize interfacial degradation, thus preventing performance losses over time. The integration of buffer layers between active and electrode layers further protects sensitive materials from external degradation factors.

Another critical innovation is the introduction of diffusion barriers, which prevent penetration of oxygen, moisture, and metal ion migration from electrodes. Ultra-thin interfacial coatings, such as atomic layer deposition (ALD) films and 2D materials like graphene, serve as effective protective layers without compromising electrical conductivity.

By implementing these engineering strategies, OSCs can achieve higher operational stability, improved efficiency, and extended device lifetimes, making them more reliable for commercial applications [132].

## 10. Applications of DFT in the Design of Materials for Organic Solar Cells

The theoretical understanding of organic solar cells (OSCs) is crucial to integrating first-principles simulations and molecular dynamics (MD) studies, particularly in the context of material degradation mechanisms. These simulations provide insights into how materials degrade under real-world conditions and, potentially, impact solar cell performance. DFT serves as a method for predicting the degradation pathways of OSC materials at the molecular level. DFT simulations effectively model the changes in electronic structure that occur under conditions like UV exposure or oxidation, which frequently contribute to the degradation of organic semiconductors. Thus, DFT can be employed to forecast the oxidation potentials of donor and acceptor materials, as well as to determine how molecular orbitals (HOMO-LUMO levels) are affected by degradation [133].

In addition to first-principles DFT, molecular dynamics (MD) simulations are a robust tool for exploring the long-term behavior of organic materials when subjected to different environmental stressors. MD simulations facilitate a detailed examination of molecular dynamics over time and space, offering a critical understanding of the morphological stability of OSCs, particularly regarding the phase separation between donor and acceptor materials [141]. Furthermore, MD simulations help understand the effects of temperature, humidity, and UV radiation on the structural integrity of OSCs.

### 10.1. Prediction of Energy Band and Molecular Energy Levels

One of the most critical factors in the design of OSC is the selection of materials with an optimal bandgap and energy level alignment (HOMO-LUMO) to ensure efficient charge transfer. The ability to calculate and predict these energy levels precisely before material synthesis is crucial for improving OSC performance and stability.

**Figure 12:**
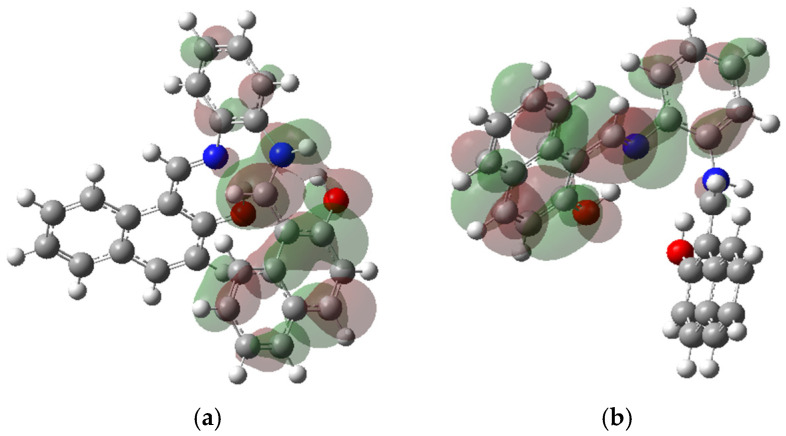
(**a**) A plot of HOMO of an electron acceptor. (**b**) A plot of LUMO of an electron acceptor (modified by reference).

Using density functional theory (DFT), researchers can determine with high accuracy frontier molecular orbitals HOMO and LUMO (see Figure 12). The image below is modified from previous research [142]. These plots provide a detailed understanding of how electrons move within systems and how well the material will function in a solar cell. The HOMO level represents the highest energy state an electron occupies before excitation, and it determines how easily material donates electrons (donor behavior). The LUMO level, which corresponds to the lowest energy state available for an electron to transition into, defines the acceptor ability of the material. The difference between HOMO and LUMO, known as bandgap, directly affects light absorption and charge transport efficiency [132].

### 10.2. Simulation of Donor–Acceptor Interactions

**Figure 13:**
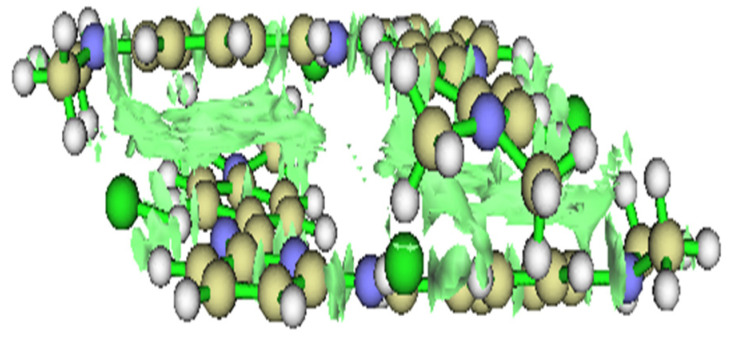
The interaction between two electron acceptors and donor molecules. The green areas indicate interaction zones through which charge carriers can pass.

The efficiency of OSCs depends largely on interactions between donor and acceptor materials, which determine how effectively charge separation and transport occur. Using density functional theory (DFT), researchers can analyze these intermolecular interactions at a fundamental level, allowing for the rational design of high-performance OSCs. Figure 13 is modified from previous research [142].

By computing the electronic properties of donor–acceptor pairs, it is possible to select materials that exhibit strong electronic coupling while minimizing recombination losses. Optimizing the molecular organization for charge transport beyond the electronic properties, DFT modeling helps optimize the structural arrangement of donor and acceptor molecules. The way these materials pack within an active layer determines charge mobility and stability. Key insights obtained from DFT include:

- π-π stacking interactions, which enhance charge delocalization and conductivity.
- Interfacial energy levels affect how efficiently electrons and holes are extracted at the electrodes.
- Molecular reorganization energies predict how easily charges move within the material.

### 10.3. Evaluation of Light Absorption

Time-dependent DFT (TD-DFT) is a powerful tool for calculating the absorption spectra of materials used in OSCs. This analysis is essential for identifying compounds with optimal absorption across the solar spectrum, which directly impacts energy conversion efficiency.

**Figure 14:**
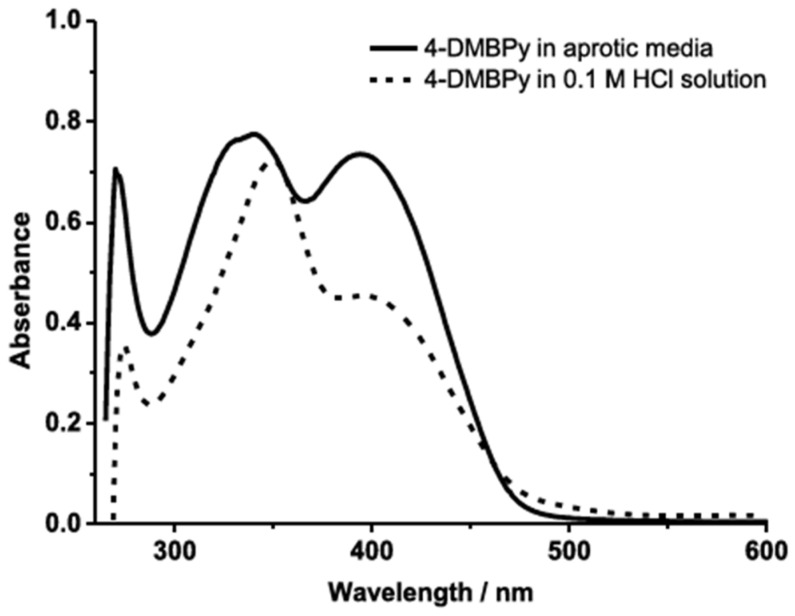
Simulated UV-V is spectrum using TD-DFT [142].

Simulating electronic transitions between molecular orbitals is shown in Figure 14. TD-DFT helps determine which wavelengths of light a material can absorb. This is crucial for designing materials that maximize solar energy utilization. Molecules that absorb longer wavelengths (near infrared or red regions) have smaller bandgaps, which facilitates the transition of charge carriers from HOMO to the LUMO. A narrower bandgap reduces energy losses, allowing for more efficient charge transport and improved photocurrent generation. Through DFT and TD-DFT modeling, material selection can be guided toward highly efficient OSCs with tailored optical properties, ensuring better light harvesting and enhanced energy conversion.

### 10.4. Charge Transport in Organic Solar Cells

Charging transport in active materials of OSCs plays a fundamental role in determining energy conversion efficiency. For an OSC to function effectively, photogenerated charge carriers must move efficiently through donor–acceptor interface and reach their respective electrodes without significant losses due to traps, recombination, or high-resistance pathways. DFT provides a powerful computational framework to model and predict charge carrier mobility, offering key insights into the design of more efficient photovoltaic materials.

One critical aspect of charge transport is the active layer’s ability to inject and extract charges efficiently. Using DFT calculations, researchers can estimate energy barriers at material interfaces, which influence how easily charge carriers (electrons and holes) move from the active layer into electrodes. A low energy barrier facilitates smoother charge extraction, reducing losses and improving the device’s power conversion efficiency (PCE).

Additionally, as shown in Figure 13, DFT helps identify molecular structures that promote strong π-π stacking interactions, which enhance charge delocalization and improve carrier mobility. Modifying molecular conjugation length and functional groups allows for fine-tuning energy level alignment between donor, acceptor, and electrode materials, ultimately optimizing charge transport properties.

## 11. Discussion

The performance and durability of photovoltaic (PV) modules are significantly influenced by the environmental conditions of the sector where they are operating, such as humidity, ultraviolet (UV) radiation, and thermal stress (see Table 1). The variations in degradation modes, exposure periods, and environmental [143] stressors promote the development of tailor-made mitigation strategies to improve the durability of PV systems in different climatic zones, for example: (i) High humidity regions such as the USA, Japan, South Korea, Germany, or the Netherlands are predominantly dominated by electrochemical and galvanic corrosion since moisture infiltration into the solar panel accelerates the oxidation of metallic contacts and encapsulant degradation, respectively. (ii) Coastal and industrial environments such as China, Spain, Italy, and Mexico exhibit photocorrosion and delamination problems, degraded by exposure to saline water and airborne pollutants. (iii) UV radiation and thermal stress-driven degradation are observed in desert environments such as Algeria, Morocco, and Saudi Arabia, resulting in encapsulant yellowing, weld failure, and loss of adhesion on backing materials [144,145]. Prolonged exposure to moisture and high relative humidity is one of the main factors contributing to the degradation of photovoltaic modules. This phenomenon causes (i) electrochemical and galvanic corrosion, which occurs at metal contacts and welds, especially in coastal environments or those with high condensation and salinity; (ii) encapsulant delamination by moisture absorption through the encapsulant (EVA or PVB), which generates bubbles and loss of adhesion to the cell and the protective glass; and (iii) degradation of insulation resistance [146] by moisture, which can generate conductive paths within the module, increasing current leakage and reducing efficiency.

Degradation behavior varies considerably and is dependent on the technology used. (i) The most studied technology is crystalline silicon (c-Si and mc-Si), which exhibits electrochemical corrosion, grid line degradation, and encapsulant delamination [147,148]. Long-term data (10–37 years) from installations in the US, Italy, and Spain indicate that corrosion of the metallization layers is a key limiting factor. (ii) Thin-film panels (TF and a-Si) are more susceptible to moisture intrusion, leading to pitting corrosion and loss of adhesion. Studies in the US and Germany suggest that thin-film modules degrade faster under high humidity conditions. (iii) Recent research on HIT and PSCs devices in Japan and China reports challenges with corrosion of metal contacts, leading to loss of electrical conductivity. The stability of these materials is a critical area of research for improving the next generation of PV systems.

The period of environmental exposure is crucial to the severity of corrosion due to the following. (i) Short-term testing (1–5 years): accelerated laboratory aging studies (USA, Japan) indicate that thermal stress testing (85/85) [55] can reliably predict long-term failure modes. (ii) Medium-term field studies (6–20 years): installations in Spain, Germany, and China suggest that modules in coastal and industrial environments degrade faster than in arid areas due to higher corrosion rates. (iii) Long-term operational data (>20 years): sites in Switzerland, Italy, and Canada show that PV modules degrade at an average rate of 0.5–1% per year, with dominant failure mechanisms being corrosion, encapsulant yellowing, and weld fatigue.

While degradation mechanisms are often studied in isolation [149,150], field performance data suggest synergistic interactions between environmental factors such as (i) UV radiation, which accelerates encapsulant degradation, increasing moisture infiltration and electrochemical corrosion of metal contacts; (ii) thermal cycling, which induces mechanical stress, leading to solder fatigue and crack propagation in encapsulant materials; and (iii) the combination of high humidity and contaminants, which favors delamination, reducing the electrical resistance of the insulation and increasing potential-induced degradation (PID), respectively.

The degradation patterns observed in Table 1 confirm that environmental conditions play a decisive role in the durability of photovoltaic modules. The interaction between humidity, UV radiation, temperature variations, and contamination underscores the need for a comprehensive approach to corrosion management. Future research should prioritize innovations in materials, advanced coatings, and real-time diagnostic systems to ensure the long-term viability of photovoltaic installations in diverse climatic regions.

Analyzing photovoltaic module degradation using advanced characterization techniques allows for precisely identifying deterioration mechanisms and their effects on long-term performance. The data in Table 1 show that electrochemical, structural, and optical characterization plays a fundamental role in evaluating the degradation mechanism under different technologies and environmental conditions. The characterization techniques used in photovoltaic module degradation studies can be grouped into different categories according to the type of deterioration analyzed. Visual and structural inspection techniques identify macroscopic defects such as delamination, discoloration, and fractures in encapsulants and cells. Scanning electron microscopy (SEM) and energy dispersive spectroscopy (EDS) help evaluate the corrosion of metallic contacts and the formation of oxidation products resulting from the photovoltaic panel operation. In turn, optical and spectroscopic techniques have been implemented to assess material aging, such as (i) X-ray photoelectron spectroscopy (XPS) to determine the chemical composition and oxidation of metallic contacts), (ii) Fourier transform infrared spectroscopy (FTIR) to detect chemical changes in the encapsulants due to UV exposure, and infrared thermography (IR) to identify areas with overheating in connections or material degradation, respectively. The combination of characterization techniques allows us to correlate different degradation mechanisms with the specific environmental factors for each location. According to the analyzed studies: (i) Electrochemical degradation of metallic contacts (galvanic corrosion, Sn and Pb migration) has been identified by (a) SEM/EDS, which shows the accumulation of corrosion products and loss of conductive material [123,128]; (b) XPS, which confirms the formation of oxides in Ag and Cu electrodes; and (c) I-V measurements, which record increases in series resistance and reduction of module efficiency, respectively. (ii) Encapsulant aging and delamination have been associated with (a) FTIR and DSC (differential scanning calorimetry) to see changes in the chemical structure of EVA and its loss of thermal stability, and (b) IR thermography, showing thermal failures caused by the loss of adhesion between layers.

Long-term exposure to UV radiation leads to negative effects on PV modules, such as the following. (i) Photodegradation of the encapsulant and the protective glass: (a) in c-Si and mc-Si modules, UV radiation causes the formation of chromophores in the EVA, resulting in yellowing and decreased light transmission; and (b) in perovskites [36], UV induces photochemical reactions that degrade the material structure, affecting the stability of the electrical performance. (ii) Accelerated oxidation of metallic contacts: (a) UV exposure contributes to the formation of silver oxides (Ag_2_O) at the interconnections and the loss of conductivity; and (b) in perovskite modules, photochemical degradation affects the stability of the gold (Au) contact, reducing its efficiency. (iii) Photocorrosion of the conductive layers, which is usually seen in thin-film modules and HIT cells: In this case, UV radiation can induce the decomposition of TCO materials (transparent conducting oxides such as ITO and ZnO), affecting charge transfer. Furthermore, the impact of UV radiation on the degradation of photovoltaic modules has been studied using (i) UV-vis spectrophotometry, which evaluates the loss of transparency of the encapsulant and the optical absorption of affected cells, and (ii) electroluminescence (EL), which identifies regions with lower charge generation due to UV-induced defects.

Advanced characterization techniques have been key to understanding the degradation mechanisms of photovoltaic modules under various environmental conditions. Combining methods has enabled the identification of the main failure mechanisms and the development of effective mitigation strategies. As the photovoltaic industry continues to evolve, the integration of real-time diagnostic techniques and artificial intelligence-based predictive models presents a promising path to improving solar systems’ long-term durability and reliability. Regarding the effect of thermal stress on photovoltaic degradation, modules experience extreme temperature variations due to day–night cycles and severe weather conditions (see Table 1). This leads to the following. (i) Material expansion and contraction: (a) in c-Si and mc-Si, thermal stress causes microcracks in the cells, reducing the energy conversion efficiency; and (b) in perovskites [37], thermal cycling can induce ion migration in the active material, leading to current generation instability. (ii) Solder fatigue and failure: (a) in conventional modules, thermal cycling accelerates mechanical fatigue at Sn-Pb and Ag interconnections; and (b) in HIT and PSCs modules, the differential thermal expansion coefficient between layers can generate internal delamination and fractures at the interface. (iii) High temperature induced degradation: (a) in perovskites [29], thermal stability is a challenge as temperatures above 85 °C can induce decomposition of the active material; and (b) in c-Si modules, the PID (potential induced degradation) effect is favored by elevated temperatures and high operating voltages.

## 12. Conclusions

Corrosion in solar panels presents a significant challenge to the efficiency and durability of photovoltaic (PV) systems, compromising their profitability and long-term viability. Through this review, it has been demonstrated that certain corrosion mechanisms, such as galvanic corrosion, are complex processes influenced by environmental factors, including humidity, temperature variations, and exposure to UV radiation and pollutants such as CO_2_, Cl^−^, SO_2_, and NO_x_, among others. The degradation models analyzed allow for the prediction of corrosion impact on PV modules but have limitations in replicating real-world conditions, highlighting the need for field validation studies. Characterization using advanced techniques such as SEM, FE-SEM, EDS, and XPS provides critical insights into corrosion progression and type, facilitating the development of effective mitigation strategies. Additionally, using first-principles simulations (DFT), it is important to predict and understand various degradation mechanisms at the atomic and molecular levels. These methods inform the development of more durable materials for solar panels, leading to more reliable and longer-lasting solar technologies.

From a sustainability and technological development perspective, advancing in the selection of corrosion-resistant materials and optimizing PV module designs to minimize degradation is essential. Additionally, the implementation of monitoring and predictive maintenance programs will enable early fault detection and a reduction in operational costs. Looking ahead, designing new materials with anticorrosive properties, developing more efficient encapsulants, and using artificial intelligence to predict and manage degradation will be key to ensuring the reliability of photovoltaic systems. Overcoming these barriers will help establish solar energy as a truly sustainable and economically viable solution in the global energy transition. 

## Figures and Tables

**Table 1:** An overview of solar panels exposed to different environments around the world.

Ref.	Technology	Degradation Modes	Temp °C	HR (%)	Irradiation (kWh/m^2^/Year)	Location	Exposure Period	Characterization Techniques
[20]	c-Si	Electrochemical and galvanic	85	2.5/70/98	-	USA	1944 h	I-V and P-V measurements Visual measurements: SEM, EDAX, ESCA, Auger Surface analysis
[38]	a-Si	Electrochemical and galvanic	85	85	-	USA	1200 h	SEM, EDX, SIMS. LSV, I vs. t, power output vs. Q
[39]	TF/a-Si	Electrochemical, galvanic, and pitting	85	85	-	USA	1910 h	LSV (E_corr_ = 500 mV and I_corr_ = 1.5 mA)
[40]	sc-Si	-	−10 to 35	<90	1393.6	Italy	19 years	Visual inspection IR I-V and P-V measurements Insulation tests
[41]	sc-Si	-	7 to 17	75 to 80	1493 (Arcata)	USA	11 years	I-V and P-V measurements
[42]	TF/a-Si	Electrochemical	60/72/85	85	-	USA	170 to 1340 h	Visual inspection BHAST
[43]	c-Si	Electrochemical, pitting, and photocorrosion	−10 to 35	<90	1393.6	Italy	19 years	I-V and P-V measurements
[44]	TF/a-Si/a-SiGe	Electrochemical	85	85	-	USA	125 h	AFM, DRX
[45]	c-Si	Electrochemical, oxidative degradation, hydrolytic reaction, and delamination	85	100	-	USA	-	Diffusivity and solubility measurements WVTR
[46]	sc-Si/mc-Si	Electrochemical, photocorrosion, and delamination	40/90	93/95	1393.6	Italy	20 to 22 years	I-V and P-V measurements Visual inspection Electrical insulation test Bypass diode check Laser scan measurements
[47]	TF	Electrochemical, galvanic, and delamination	60/85/95	60/85/100	-	USA	19 to 967 h	Xenon arc lamp radiation test Damp-heat test Adhesion measurements Scotch tape adhesion test XPS
[25]	c-Si	Electrochemical and galvanic	-	-	-	Spain	-	-
[48]	a-Si/c-Si/mc-Si	Electrochemical, photocorrosion, and delamination	25	-	2054.5	USA	1 to 7 years	Visual inspection I-V and P-V measurements IR
[49]	c-Si	Electrochemical, photocorrosion, and delamination	60	60	-	USA	6.25 months	Lap shear tests UV exposure tests Accelerated stress chamber tests Transmittance measurements Water absorption measurements
[50]	c-Si	Electrochemical, photocorrosion, and delamination	18	63	1890	Spain	12 years	Visual inspection I-V and P-V measurements IR Spectrophotometry
[51]	c-Si	Electrochemical and photocorrosion	85	-	-	Rusia	1000	UV radiation exposure test Spectral transmittance measurement Visual inspection I-V and P-V measurements
[52]	-	Electrochemical, general corrosion, and delamination	85	85	-	Germany	1000	Visual investigations EL Wet leakage testing Peel strength measurement Bulk resistivity measurements
[53]	sc-Si	-	16 to 38	75 to 95	2114	Senegal	1 year	I-V and P-V measurements
[54]	c-Si	General corrosion	~7.5	~86.5	-	France	2700	-
[55]	mc-Si	Galvanic corrosion	45/65/85	85	-	-	4000	SEM, EDX, AES, EL
[56]	a-Si, mc-Si HIT	General corrosion	11 to 34	42 to 62	1621	India	1 year	I-V and P-V measurements
[57]	c-Si	Electrochemical	45/65/85	85	-	USA	1 year	I-V and P-V measurements Moisture weight gain measurements Solubility determination WVTR
[58]	c-Si	Galvanic corrosion	85	85	-	Republic of Korea	3000	SEM, EDX, EL
[59]	sc-Si/mc-Si	Electrochemical	16 to 38	75 to 95	2114	Senegal	16 to 48 months	I-V and P-V measurements
[60]	c-Si	Chemical corrosion	85	85	-	Japan	4000	EL, EPMA, FTIR, SCM
[61]	a-Si	Electrochemical	25	-	-	Egypt	-	I-V and P-V measurements
[62]	sc-Si	Electrochemical and galvanic corrosion	20 to 25	80	1517.6	India	28 years	Visual inspection IR I-V and P-V measurements
[63]	Si	Electrochemical	13.6	64	1515.4	Republic of Korea	6 years	FE-SEM, EDS, FIB,
[64]	sc-Si	-	16 to 38	75 to 95	2114	Senegal	1 year	I-V and P-V measurements
[65]	c-Si	Electrochemical and galvanic corrosion	6 to 28	19 to 45	2014.8	USA	6–16 years	Visual inspection IR I-V and P-V measurements
[66]	c-Si	Electrochemical	85	85	-	The Netherlands	2000 h	Visual inspections I-V and P-V measurements
[67]	sc-Si	Electrochemical	7 to 42	19	2209.7	Algeria	-	Visual inspection I-V and P-V measurements
[68]	mc-Si		24.4 to 27.8	65 to 83.5	1725 (Kumasi)	Ghana	19 years	Visual inspection IR I-V and P-V measurements
[69]	sc-Si	Electrochemical and photocorrosion	47.4	16 to 52	2066.5	Algeria	12 years	Visual inspection I-V and P-V measurements Analytical calculations of degradation rates Insulation resistance measurements Cost analysis
[70]	sc-Si	Electrochemical	9 to 44	10 to 40	2209.7	Algeria	>12 years	Visual inspection I-V and P-V measurements
[71]	mc-Si	Grid corrosion, galvanic	25	45/85	-	China	240 h to 2 months	EL I-V characteristic measurement SEM EDS
[72]	c-Si	-	10 to 35	90	1393.6	Italy	20 years	Visual inspection Electrical performance measurements EL IR
[73]	sc-Si/mc-Si	General corrosion	20 to 28	~70	2114	Senegal	10 years	Visual inspection EL IR
[74]	c-Si/TH	Electrochemical, photocorrosion, and pitting	14 to 34	-	1764.9	India	-	Visual inspection Thermal image
[75]	c-Si	-	−0.4 to 19.7	70 to 80	1393.2	Switzerland	35 years	I-V and P-V measurements Visual inspection Electrical insulation (INS) test EL IR
[76]	sc-Si	Electrochemical, galvanic, photocorrosion, and general corrosion	14.2 to 28.3	67 to 80	1470.3	China	18 years	I-V and P-V measurements EL SEM EDX XPS
[77]	a-Si/sc-Si	Electrochemical	2.5 to 19	58 to 92	1072.5	Germany	5 years	I-V and P-V measurements
[78]	c-Si	Electrochemical and galvanic	72/85	85/95	-	USA	292 to 1000 h	SEM EDS Pourbaix diagrams
[79]	c-Si	Electrochemical and photocorrosion	25 to 120	-	-	UK	-	Finite element method (FEM) Dynamic mechanical analysis (DMA)
[80]	c-Si	Chemical and galvanic corrosion	85	85	-	Japan	7203	DH test Optical measurements. I-V and P-V measurements EL Ion chromatography
[81]	c-Si	Electrochemical and photo-corrosion	26 to 28	82 to 86	1637	Singapore	7 years	I-V and P-V measurements
[82]	sc-Si	Electrochemical and pitting	23 to 27	70–90	2003.2	Indonesia	6/8/12 years	Visual inspection
[83]	c-Si	-	25	-	-	Lithuania	-	Visual inspection EL
[84]	mc-Si	Electrochemical	25	-	-	Japan	-	Visual inspection I-V and P-V measurements EL Impedance spectroscopy Statistical evaluation
[85]	a-Si	-	9 to 44	10 to 40	2209.7	Algeria	14 months	Visual inspection I-V and P-V measurements
[86]	c-Si/a-Si	-	12.2 to 31	28.4 to 31.5	2099.6	Morocco	6 years	I-V and P-V measurements
[87]	c-Si	-	85	85	-	USA	4200 h	EL Feature extraction algorithms Hierarchical clustering Principal component analysis (PCA)
[88]	sc-Si	-	24 to 28	76.5 to 88.3	1733	Ghana	13 years	Visual inspection I-V and P-V measurements IR
[89]	c-Si	Atmospheric corrosion and pitting corrosion	-	-	-	Portugal	-	I-V and P-V measurements
[90]	sc-Si	-	30 to 85	50 to 85	-	Austria	1000 to 3000 h	I-V and P-V measurements EL Visual inspection FTIR Differential scanning calorimetry (DSC) Thermal expansion behavior characterization Thermally extractable components analysis
[91]	sc-Si	-	6 to 30	73 to 80	1289.6	China	2 years	SEM XRS
[92]	sc-Si	-	19.6	76.5	1675.5	Brazil	15 years	EL I-V and P-V measurements Visual inspection Thermography Electrical insulation test
[93]	sc-Si/mc-Si/TF	-	19.2 to 28.9	45 to 81	1645	Bangladesh	5 years	Visual inspection I-V and P-V measurements
[94]	c-Si	-	7.3 to 28.6	-	2141 (Amman)	Jordania	0 to 4 years	EL I-V and P-V measurements
[95]	mc-Si	-	15 to 31	-	1731 (Lucknow)	India	2 to 5 years	Portable monocular metallurgical microscope Photoluminescence (PL)
[96]	sc-Si	-	95	95	-	Japan	2664 h	FE-SEM EDX FIB ICP-OES
[97]	mc-Si	-	~29	~78	1843 to 2005	Thailand	15 years	Visual inspection I-V and P-V measurements EL Electrical insulation test
[98]	c-Si	-	75 to 85	85	-	USA	3 months	XPS Elemental depth profiling Optical microscopy
[99]	sc-Si	-	−10 to 21	67 to 80	1140 (Quebec)	Canada	23 years	Visual inspection Solar simulator I-V Curves Auger electron spectroscopy
[100]	mc-Si	-	12.4 to 38.6	10 to 40	2209.7	Algeria	1 year	I-V and P-V measurements
[101]	c-Si	-	85	85	-	China	1000 h	I-V and P-V measurements Dynamic load + shearing sequence test Distortion experiment (module distortion + DH2000 h) Long-term PID test
[102]	c-Si	-	85	85	-	UK	5350 h	Visual inspection EL I-V and P-V measurements Spectral response (SR) measurements
[103]	mc-Si	-	-	-	-	Republic of Korea	5 years	Visual inspection EL I-V and P-V measurements
[104]	mc-Si/sc-Si/CIS	-	26 to 28	82 to 86	1637	Singapore	10 years	Visual inspection I-V and P-V measurements EL
[105]	c-Si	-	10 to 35	90	1393.6	Italy	37 to 39 years	XRF FTIR DSC DLTMA
[106]	c-Si	-	85	85	-	China	1000 h	I-V and P-V measurements Visual inspection
[107]	sc-Si	-	10.9 to 28.2	44 to 74	1845 (Sevilla)	Spain	22 years	Visual inspection IR EL I-V and P-V measurements
[108]	sc-Si/mc-si/a-Si	-	8 to 31	31 to 57	1949 (Beni Mellal)	Morocco	4 years	I-V and P-V measurements
[109]	c-Si/HIT/CIS	-	16.3 to 38.2	16 to 52	2081 (Ghardaïa)	Algeria	1 year	I-V and P-V measurements
[110]	c-Si/HIT	-	20 to 60	-	-	Switzerland	3000 h	Visual inspection SEM EDX Raman spectroscopy
[111]	mc-Si/sc-Si/a-Si	-	−2 to 28	57 to 78	1890 (Meknes Tafilalet)	Morocco	6 years	I-V and P-V measurements Linear regression analysis
[112]	mc-si	-	26.7 to 38	-	2200	Djibouti	9.5 years	Visual inspection IR UVFL
[19]	mc-Si	Galvanic corrosion	60	55.75 and 85	-		20 years	Thermodynamic and kinetic analyses Finite element analysis
[113]	mc-Si/m-Si	Metallization corrosion and encapsulant discoloration	85	85	-	Thailand	30 years	I-V measurements Electroluminescence SEM EDS XPS
[114]	c-Si	Metallization	25	-	1000 W/m^2^	India	10 years	I-V measurements Visual inspection Electrical characterization
[115]	poly-Si	Metallization corrosion	38	-	2400	India	10 years	I-V measurements Visual inspection
[116]	c-Si	-	10	-	-	Italy	20 years	I-V and P-V measurements PV roofs
[117]	a-Si	Electrochemical corrosion	90	85		USA	6 to 7 years	
[118]	a-Si/μc-Si	General corrosion	45	-	-	Africa	5.5 year	Visual inspection P-V measurements Installation costs
[119]	c-Si	General corrosion	−10 to 50	-	1995.9	USA	-	Easy installation Low maintenance cost Reduction in CO_2_ emissions
[120]	mc-Si	Metal grid oxidation and corrosion	10 to 12	-	-	Norway	20 years	I-V measurements Visual inspection IR EL UV-F
[121]	poly-Si	Electrochemical corrosion	25	-	1000 W/m^2^	Ghana	20 to 25 years	I-V and P-V measurements Visual inspection
[120]	mc-Si	Metal grid oxidation and corrosion	10 to 12	-	-	Norway	20 years	I-V measurements Visual inspection IR EL UV-F
[121]	poly-Si	Electrochemical corrosion	25	-	1000 W/m^2^	Ghana	20 to 25 years	I-V and P-V measurements Visual inspection
[122]	poly-Si/m-Si/a-Si	General corrosion	-	75	-	Ghana	5 years	I-V measurements Visual inspection
[123]	mc-Si	Electrochemical corrosion	25		960 to 1040 W/m^2^	Norway	20 years	I-V measurements EL UV-F IR-T
[124]	c-Si	-	25	-	800–1200 W/m^2^	China	10 years	
[125]	m-si	General corrosion	−45 to 85	-	1 kW/m^2^	Andaman/Nicobar Islands	10 years	Visual inspection
[126]	mc-Si	-	25	80 to 87	1000 W/m^2^	Nigeria		I-V and P-V measurements Electrical parameters
[127]	c-Si/mc-Si	-	-	-	-	India	12 to 18 years	I-V measurements FMEA
